# Action of the Euclidean versus projective group on an agent’s internal space in curiosity driven exploration

**DOI:** 10.1007/s00422-024-01001-1

**Published:** 2025-01-17

**Authors:** Grégoire Sergeant-Perthuis, Nils Ruet, Dimitri Ognibene, Yvain Tisserand, Kenneth Williford, David Rudrauf

**Affiliations:** 1 LCQB Sorbonne Université & OURAGAN team, Inria Paris Paris, France; 2 CIAMS, Université Paris-Saclay, Orsay & Université d’Orléans, Orléans, France; 3 Department of Psychology, University of Milano-Bicocca, Piazza dellÁteneo Nuovo, 1-20126, Milan, Italy; 4 Institute of Cognitive Sciences and Technologies, National Research Council, Rome, Italy; 5 CISA, University of Geneva, Geneva, Switzerland; 6 Department of Philosophy and Humanities, The University of Texas at Arlington, Arlington, USA

**Keywords:** Geometric world model, Exploration, Embodied cognitive science, Cognitive modeling, Perception-action coupling

## Abstract

According to the Projective Consciousness Model (PCM), in human spatial awareness, 3-dimensional projective geometry structures information integration and action planning through perspective taking within an internal representation space. The way different perspectives are related to and transform a world model defines a specific perception and imagination scheme. In mathematics, such a collection of transformations corresponds to a ‘group’, whose ‘actions’ characterize the geometry of a space. Imbuing world models with a group structure may capture different agents’ spatial awareness and affordance schemes. We used group action as a special class of policies for perspective-dependent control. We explored how such a geometric structure impacts agents’ behaviors, comparing how the Euclidean versus projective groups act on epistemic value in active inference, drive curiosity, and exploration. We formally demonstrate and simulate how the groups induce distinct behaviors in a simple search task. The projective group’s nonlinear magnification of information transformed epistemic value according to the choice of frame, generating behaviors of approach toward objects with uncertain locations due to limited sampling. The Euclidean group had no effect on epistemic value: no action was better than the initial idle state. In structuring a priori an agent’s internal representation, we show how geometry can play a key role in information integration and action planning. Our results add further support to the PCM.

## Introduction

In artificial agent learning and control, intrinsic and extrinsic rewards can be combined to optimize the balance between exploration and exploitation. Intrinsic rewards in Reinforcement Learning (RL) (Hester and Stone 2017; Merckling et al. 2022; Oudeyer et al. 2007) or terms of epistemic value in active inference (Friston et al. 2015) have been suggested as mechanisms that might account for curiosity and exploratory drives, e.g. by integrating prediction error or uncertainty in order to drive actions favoring their reduction. However, efficient exploration is a computationally hard task. Recent neural planning models have increased planning flexibility and generality (Sekar et al. 2020). Yet, it is well known that models’ structures heavily impact planning performance and tractability (Geffner and Bonet 2013) as well as learning complexity (Goyal and Bengio 2022). A good representation of information may improve learning and search efficiency.

These issues are particularly salient for computation-heavy, highly recursive machine learning algorithms and applications, e.g., reinforcement learning (RL) in artificial agents (Bonet and Geffner 2019) or recursive modeling methods (RMM) in multi agent systems (MAS) and partially observable stochastic games (POSG) (Geffner and Bonet 2013).

Although generic neural world models can support explo ration-related processes, incorporating prior knowledge that shapes internal representations to more effectively support exploration across a broad range of environments, such as 3-D environments, may enable autonomous agents to explore more complex and realistic settings on a larger scale (Goyal and Bengio 2022). The exploration planning problem can thus be approached by considering how the structure of representation impacts exploration behaviors. In this article, the structure of representations is encoded into the geometry of the state space of an agent; we quantify the impact of changing this geometry on the behavior of the agent.

Here, we do not consider mechanisms of representation learning, e.g., in which world dynamics and action effects need to be learned and represented, as typically done in RL. We consider control and execution in agents with an a priori, encoded by a generative model (POMDP), on the temporal evolution of their environment conditioned on their actions. In particular, an agent has an a priori on how its observations are related to the state of its environment, and on the consequences of its actions on the environment. However, the agent is uncertain about the exact state of the environment and updates its belief about this state through successive observations. We focus on action selection for environment exploration and mapping.

We adopt the active inference framework, i.e., an implementation of the Bayesian Brain Hypothesis aimed at generating adaptive behaviors in agents (Friston et al. 2006), which has found applications in neuroscience (Da Costa et al. 2020) and was proposed for modeling molecular machines (Timsit and Sergeant-Perthuis 2021; Timsit et al. 2021). It relies on an internal representation of the environment that an agent is driven to explore and exploit. The agent continually updates its beliefs about plausible competing internal hypotheses about the state of its environment. Under common sensory limitations, active inference relates to optimal control (Kaelbling et al. 1998; Ognibene et al. 2019).

The epistemic value of states is a quantity that arises in active inference (Friston et al. 2015). Its maximization drives the agent’s curiosity and actions.

For exploration or search in 3-D space, it is warranted to consider how geometrical principles could be embedded into efficient control mechanisms in order to regularize the internal representation of information and mediate exploration under a drive of uncertainty reduction or information maximization. Geometrical considerations have previously been integrated into a variety of optimization and machine learning approaches, such as RL, active inference, and Bayesian inference (see Related Works below), but not in the specific perspective we introduce here.

We build upon the hypothesis that 3-D internal representations of space in agents performing active inference may correspond to specific geometries, with properties that can be exactly analysed. More specifically, we consider how different first-person perspectives may relate to each other, through transformations of a world model, as a specific perception and imagination scheme for agents. This entails considering the action of geometrical groups of transformations (in the mathematical sense of the concept in Group theory; see Sect. 3.1) on the spatial distribution of information experienced and encoded by agents internally. The question is whether such group action could contribute to information gain estimation and maximization, as an internal planning or perspective-dependent control mechanism. Certain geometrical groups might imply internal representations, policies, value functions and principles of action that are particularly relevant for search and exploration. More specifically, we wish to compare how different groups impact the quantification of epistemic value. We then wish to characterize how the optimization of action from those different groups may yield different exploration behaviors. We contrasted two separate toy models of an agent performing a simple search task using active inference based solely on epistemic drives. One model used Euclidean geometry and the other projective geometry for the agent’s internal space. We compared the two models in terms of resulting exploration behaviors and effects on epistemic value.

We chose to compare Euclidean versus projective geometry based on previous work, leveraging psychological research on the phenomenology of spatial consciousness and its role in the control of behaviors (Rudrauf et al. 2017b, 2020b, 2022b, 2023). This research suggested that 3-D projective geometry plays a central role in human cognition and decision-making by shaping information representation and subsequent drives. It also offers a mechanism able to account for changes of points of view and perspectives on a world model, including for perspective taking in social cognition, which is critical for the development of strategies of action planning in humans (see Rudrauf et al. 2022b, 2023). We used Euclidean geometry as a standard baseline geometry for comparison (Ognibene and Demiris 2013). Our geometrical rationale implies a different understanding of how agents’ actions in their environment (here in the behavioral sense of the term) are implemented and selected compared to usual active inference. Agents’ actions, such as navigation and approach-avoidance behaviors, can naturally be seen as dual to internal changes of perspective, i.e., group actions, in their representation space. We thus used group actions as a predictive model of actual behavioral actions.

The approach allowed us to formally study and demonstrate how the geometry governing the internal representation space may directly impact the computation of epistemic value and ensuing exploratory behaviors. Projective geometry versus Euclidean geometry demonstrated remarkable properties of information integration for motion planning under epistemic drive.

## Related Work

### Representation of Space and Exploration, in the Context of Machine Learning and Control

The integration of geometrical mapping in machine learning has been proposed to reduce the high-dimensionality of input spaces and provide efficient solutions for action selection and navigation. Seminal neurally inspired models used projections on 2-D manifolds for representation learning of complex spatial information and self-motion effects (Arleo et al. 2004). The impact of changes of perspective in exploration has long been of interest (Ognibene and Demiris 2013). Ferreira et al. (2013) proposed an internal 3-D egocentric, spherical representation of space, to modulate information sampling and uncertainty as a function of distance, and to control a robot attention through Bayesian inference. This was a seminal example of how a geometrical rationale could suggest solutions to the problem of integrating perception and action planning.

Methods of exploration must often maintain high-resolution representations of a space to maximize information gain following action. This may hinder exploration efficiency, in particular in large-scale environments. 3-D topological representations of ambient space have been proposed as part of an abstract planning scheme, showing promising improvements in exploration efficiency (Yang et al. 2021).

Active vision principles, combined with curiosity-based algorithms and RL, were applied to the learning of saliency maps in the context of autonomous robot navigation (Craye et al. 2016). The approach yielded promising optimization solutions to both adaptive learning of task-independent, spatial representations, and efficient exploration policies, which could serve as priors to support long-term, task-oriented, utility-driven RL mechanisms (Craye et al. 2016) (see also Ognibene and Baldassare 2014; Sperati and Baldassarre 2017).

Information theoretic methods have been widely explored in the modelling of gaze behaviours (Bruce and Tsotsos 2005), however the role of the internal space adopted has received less attention.

Complex control problems with continuous state and action spaces have been solved using deep reinforcement learning (DRL) with joint learning of representations and predictions. Such approaches may entail non-stationarity, risks of instability and slow convergence, in particular in control tasks with active vision. Separating representation learning and policies’ computations may mitigate the issues, but it may also lead to inefficient information representations. Merckling et al. (2022) have sought to build compact and meaningful representations based on task-agnostic and reward-free agent-environment interactions. They used (recursive) state representation learning (SRL) while jointly learning a state transition estimator with near-future prediction objectives in order to contextually remove distracting information and reduce the exploration problem’s complexity. Positive outcomes were maximized through inverse predictive modeling, and prediction error was used to favor actions reducing uncertainty, which improved subsequent performance in RL tasks. The authors emphasized that dealing with partial observability through memory and active vision may require new solutions to both the representation learning of hidden information and exploration strategies.

Uncertainty-based methods using intrinsic reward and exploration bonuses in order to plan trajectories have been criticized for inducing non-stationary decaying surprise and for being hard to structure and optimize (Guo et al. 2021). Maximum State-Visitation Entropy (MSVE) was introduced to maximize state exploration uniformity, but optimization has often been challenging for large state spaces. Guo et al. (2021) have introduced Geometric Entropy Maximization, which leverages geometry-aware entropy based on Adjacency Regularization (AR) and a similarity function, in order to optimize the MSVE problem at scale.

Geometrical constraints considered across these related works were not integrated into a global model and were somewhat ad hoc. They pertained to a lower level of processing than the one we are concerned with here. However, they emphasize the current needs and challenges for integrating geometry into learning, control and navigation.

Methods and algorithms combining computer vision, machine learning and optimization, e.g., for robotic planning, have integrated group theoretic concepts to obtain, for instance, invariance to rotation and translation in image processing (Lee and Moore 2004; Qin et al. 2019; Meng et al. 2017). Likewise, the leveraging of geometrical concepts, in Deep learning, e.g., for learning manifolds and graphs, has been growing in recent years, demonstrating very promising results for representation learning (Gerken et al. 2021; Cao et al. 2022). The approach introduces combinatorial structures in order to leverage prior knowledge of geometry for dealing with the data of interest, e.g., applying ‘convolutional Neural Networks’ to non-Euclidean space. However, the Euclidean group $E_3$, or more specifically $SE_3$ (see Lee and Moore 2004), which includes translations and rotations, but excludes reflections, or simply $SO_3$, the 3-dimensional rotation group (Gerken et al. 2021), are the groups typically under consideration.

Here, in addition to the Euclidean group, we also consider $PGL_4({\mathbb {R}})$, the projective general linear group which acts on the 3-D projective space through projective transformations. The projective group is central to computer vision, for instance to generate 2-D images from 3-D information, but is used in such contexts in a restricted manner. We sought approaches based on cognitive science, considering spatial cognition and its relations to action at a higher level of integration, which does not reduce to the visual modality, but instead assumes the mapping of multimodal information onto a supramodal internal space of representation.

### Projective Consciousness Model (PCM) and Active Inference

#### General principles

The Projective Consciousness Model (PCM) shows that geometrically constrained active inference can be used as a framework for understanding and modeling central aspects of human spatial consciousness and its relations to behaviour (Rudrauf et al. 2017b, 2022b). According to this model, consciousness accesses and represents multimodal information through a Global Workspace (Dehaene et al. 2017) within which subjective perspectives on an internal world model can be taken. The process supports the ability to appraise possible actions based on their expected utility and epistemic value (Rudrauf et al. 2022b). In publications on PCM (Rudrauf et al. 2017b; Williford et al. 2018; Rudrauf et al. 2022b, 2023; Williford et al. 2022), it was hypothesized that such an internal representation space is geometrically structured as a 3-D projective space, denoted $P_3({\mathbb {R}})$. A change of perspective then corresponds to the choice of a projective transformation $\psi$, i.e., the action of an element of the group of projective transformations $PGL_4({\mathbb {R}})$. A projective transformation is a linear isomorphism $M_\psi \in GL_4({\mathbb {R}})$ up to a multiplicative constant. The model yielded an explanation for the Moon Illusion (Rudrauf et al. 2020b) with falsifiable predictions on how strong the effect should be depending on context; as well as for the generation of adaptive and maladaptive behaviors, consistent with developmental and clinical psychology (see Rudrauf et al. 2022b). Though essential in integrative spatial cognition, notably for understanding multi-agent social interactions, perspective taking is rarely integral to existing models of consciousness or formally implemented (Koch et al. 2016; Kleiner and Tull 2021; Mashour et al. 2020; Dehaene et al. 2017; Merker et al. 2022b). The PCM assumes that projective mechanisms of perspective change are integral to the global workspace of consciousness, both in non-social and social contexts. The advantages of mechanisms of perspective taking for cybernetics remain to be fully formulated (see Rudrauf et al. 2022b).

#### Elements of motivation for the PCM implication of 3-dimensional projective geometry in integrative, multimodal spatial perception, cognition and action planning

The PCM offers a robust framework for understanding the integration of multimodal sensory information, (e.g., vision, audition, and proprioception) and action planning within a unified spatial representational format.

Importantly, when considering perception and imagery or imagination, e.g., visual perception, one considers the phenomenology of experience, as accessed in awareness following sensorimotor processing, through still ill-defined mechanisms, which are typically studied with psychophysical methods or experimental phenomenology. We are dealing with an “internal" 3-dimensional, virtual space, a robust “controlled hallucination” of sorts, which entails some form of a top-down, a priori structure that cannot be simply or directly reduced to the “external” ambient space, nor to bottom-up sensory processing or specific constraints of sensory organs, e.g., in the case of vision to optical projections onto the retina or “optic array” (Koenderink 2012, 2013; Koenderink and van Doorn 2008). Multiple layers of abstraction or virtualization are involved (Rudrauf 2014).

According to Hering’s Law, 3-dimensional vision appears to be structured through 3-dimensional lines of sight passing through a virtual “cyclopean” eye located between the two eyes and an origin or “egocentre” (a technical term from psychophysics), typically located 4-5 cm behind the bridge of the nose (Hering 1879/1942); see also (Merker et al. 2022a; Crawford et al. 2011). The structure of the visual space itself has been studied for more than a century, with results that are sometimes difficult to interpret, in part due to complex and indirect methodologies. Results from series of studies aimed at testing Luneburg (1950)’s classical hyperbolic theory suggest that the visual space is complex and dynamical, and cannot be accounted for by a constant curvature geometry (hyperbolic, elliptical or Euclidean) with a fixed relation to physical space (Koenderink 2016; Koenderink et al. 2010b; Koenderink and van Doorn 2008; Doumen et al. 2006; Cuijpers et al. 2001). It may manifest some hyperbolic or Euclidean features depending on sensorimotor contexts and relative distance (Indow 1991, 1997; Koenderink et al. 1996), might not be isotropic according to exocentric pointing tasks (van Doorn et al. 2013); see also: (Doumen et al. 2006), and may violate fixed basic linear perspective (Koenderink 2016).

However, such variations do not override the overall 3-dimensional projective geometrical perspectival structure of visual space (Erkelens 2015; Koenderink et al. 2002).

Furthermore, a fixed overt direction of gaze in a constant environment does not entail a fixed perspective in visual awareness. There are processes of perceptual inference inducing covert changes in the viewpoint of the “mental eye" and “mental perspective”. This is for instance the case in “pictorial relief,” in which a virtual sense of depth and 3-dimensional perspective from 2-dimensional pictures is automatically generated based on depth cues (Koenderink et al. 2001). Likewise, such mental direction of sight can be actively directed up to 20 degrees to analyse the content of the visual field from different virtual local perspectives (Koenderink et al. 2015). This ability might be key to relate snapshots of visual awareness to the larger visual space as can be sampled through eye, head and body movement. The transformation group proposed for modeling pictorial space (Koenderink 2012; Koenderink and van Doorn 2008) is contained in the group of transformations entailed by the PCM. Pictorial relief experiments suggest that the visual experience of directions can be more Euclidean (parallel lines appear more parallel) than expected from retinal projections of “optic arrays” (Koenderink et al. 2010a). This is fully compatible with the PCM, which is based on 3-dimensional projective geometry, dealing with projective space (vs. 2-dimensional projective geometry, which deals with the projective plane). In projective space, parallel lines will appear more and more parallel as the location of the plane at infinity is moved farther away toward “actual” infinity.

Furthermore, the PCM assumes that the geometry of subjective space is under an ongoing process of calibration and inference, related to the intrinsic ambiguity of sensory information, which modulates the 3-dimensional projection, its projective frame and metrics, as a function of individuals, contexts and contents. In that spirit, the PCM could account for pictorial relief and several other dynamical visual illusions such as the mentioned Moon Illusion and Ames Room Illusion (Rudrauf et al. 2020a).

The PCM’s assumptions about spatial perception and cognition are consistent with the overall structure and dynamics of visual awareness and can accommodate several of the complex modulations of its phenomenology. However, the PCM’s principles are not based strictly on vision, and there are many detailed specificities of visual spatial phenomenology that the PCM does not aim to capture and that require the addition of other mechanisms. The PCM aims at modeling spatial consciousness and its subjective perspective as a multimodal or supramodal or amodal phenomenon in the context of representation and action. The scientific literature on the topic is rich but relatively limited regarding the specific question of the role of 3-dimensional projective geometry in such a multimodal phenomenology.

To begin with, it is a mistake to consider visual awareness as a phenomenon purely and uniquely related to visual organs (the eyes) and strictly visual pathways. It is textbook knowledge that normal visual awareness depends, at the least, on vestibular information (which contributes to encoding the position of the head in space and self-movement) and proprioceptive information (which contributes to encoding the body position and movement in space) (Cullen and Taube 2017; DeAngelis and Angelaki 2012; Brandt et al. 2014; Brandt 2013; Borel et al. 2008; Berthoz 2002). Those are fine-tuned to stabilize gaze and the representation of the main axes of the body, enabling rapidly adapting reference frames, for robust orientation in space and navigation, in spite of the variability of sensory inputs.

Evidence suggests that multiple sense modalities (vision, audition, proprioception, touch) may contribute to spatial perception and cognition through their integration into a global common space, instead of remaining in “disjunct spaces” requiring translations between them (Auerbach and Sperling 1974; Marks 1978); even though they may have, when considered in isolation, their own reference frames and metrics. More generally, human perceptual systems tend to favor cardinal directions (vertical, lateral and horizontal) related to natural axes of the body, sensory organ configurations, and vestibular gravity sensing, with a strong bias for egocentric reference frames (see Volcic et al. 2007). In other words, telemetric sensing (vision and audition) and orientation and pointing are conditioned by similar spatial constraints externally, which may well favor, at least partially, a unified integrative, adaptive representation of space. The integration of multimodal information into a coherent, unified subjective impression of space relative to the observer and within the environment might be necessary for optimally locating stimuli, coordinating eye movements and goal-directed actions, e.g., reaching, and, more generally, for combining visual motion, eye movement and vestibular information to support navigation and ensure perceptual stability during motion (Andersen 1997).

In addition to the visual egocentre, an auditory egocentre has been demonstrated (Sukemiya et al. 2008), which is located around the midpoint of the interaural axis (about the centre of head rotation) in congenitally blind subjects and fused with the visual egocentre in late-onset blind and blindfolded normally sighted subjects, for frontal sound sources. When sound sources are located outside the frontal field, the auditory egocentre tends to move backward to a position similar to that observed in congenitally blind subjects (Neelon et al. 2004). Thus the setting of reference frames for the multimodal 3-dimensional perception of space is a dynamical process depending on the potentially or actually available information from specific sensory modalities for optimal sampling; and when consistent multimodal spatial information can be sampled simultaneously, a common multimodal reference frame may be set.

Beyond perceptual inference, congenitally and early blind subjects use spatial imagery (Brambring 1976). They are capable of perspective taking, and use it for planning, e.g., using verbal descriptions, motion, or auditory signals for self-positioning in space; even though they may be less accurate (see Tinti et al. 2018). Comparable performances have been demonstrated in congenitally blind, late blind and blindfolded normally sighted subjects, when drawing arrays of 3-dimensional objects from different imagined standpoints, respecting configurations, shape and metrical properties (Heller and Kennedy 1990), and when spontaneously performing spatial perspective taking in order to describe the relative direction of target buildings from the perspective of another person (Tinti et al. 2018).

Blumenfeld (1937) held that the geometrical laws governing haptic and visual spaces are identical, and showed (however with some methodological limitations) that haptically constructed parallel lines along the median plane tended to diverge towards the subjects, which is consistent with a perspectival structure of the haptic space. Pick (1974) held that the sighted normally recode tactual impressions into visual images when touching forms (see also Révész 1950). In any case, the haptic space appears not to be Euclidean (Kappers and Koenderink 1999), and might involve the dynamical calibration of an intermediary reference frame combining egocentric and allocentric frames, with substantial interindividual variability (Volcic et al. 2007). Inconsistent haptic and visual perception of surface curvature can coexist, even though vision tends to dominate in such sensory conflicts, but haptic inputs can also help to disambiguate and interpret visual information depending on context (Wijntjes et al. 2009; Kappers et al. 1997; Ernst and Banks 2002). In the absence of visual information, haptic information is sufficient for estimating the position and size of objects and for guiding action; and when combined with visual information, haptic information contributes to better reaching performance (Camponogara and Volcic 2021). The hypothesis of the prevalence of a remapping onto the visual space over mutual remappings across modalities is still debated but is apparently often observed; and there is also evidence of supramodal or amodal representations of space integrating multimodal information. This is, for instance, suggested by results related to cross-modal interactions or multi-sensory integration in the context of sensory conflict, in which the visual space tends to impose its structure on other modalities to maintain a coherent experience. There are extensive spatial relationships between modalities and crossmodal interactions, including through attentional mechanisms. This includes evidence for both preattentive and voluntary attentional shifts based on multimodal internal representations of space, enabling the mutual remapping and driving of covert attention by touch, audition, vision, or proprioception, in a manner that improves information localization performance but can also lead to cross-modal auditory-visual illusions such as ventriloquism and the McGurk Effect (see Driver and Spence 1998). Multisensory localization conflicts often result in illusions of bodily positions being shifted, such as the Rubber Hand Illusion, in which touch and proprioceptive information about the localization of one’s hand are somehow remapped to be consistent with visual information (Botvinick and Cohen 1998), or in dissociations between the experienced position of the full body in 3-dimensional space and the visual first-person perspective, such that subjects can, for instance, feel that their body is located in front of them (see Blanke 2012).

Such results show that multisensory information tends to be integrated into a common experiential space, somewhat dominated by a vision-like frame of reference and that preserving the coherence of the overall space takes priority for the brain over maintaining the normal consistency of its contents. In the purely visual domain, illusions such as the Ames Room Illusion also show that maintaining size constancy is secondary to satisfying priors about the overall structure of perceived space (see Rudrauf et al. 2020a). The Ames Room Illusion, full-body multisensory illusions, and other related phenomena can be well explained by the PCM and the role of 3-dimensional projective geometry in the overall awareness of space (Rudrauf et al. 2017a, 2020a).

Perceptual inference, action planning and control within “psychological space” rely on the robust selection and interaction of multiple (gaze-centered, head-centered, body-centered, limb-centered) egocentric and (object-centered, world-centered) allocentric reference frames and spatial transformations between them to relate sensory systems, effectors and action target locations (Fiehler and Karimpur 2023; Neely et al. 2008). Dynamic selection of frames depends on the scope of motion, spatial scales (from small-scale finger movements or figural spaces, to vista spaces, environmental spaces and map-based geographical spaces), constraints from high-level cognitive factors (memory, tasks, semantics), available sampling from specific sensory modalities, and a dynamical calibration of the boundaries and metrics of the so-called peripersonal and extrapersonal spaces, which might be better conceived as a graded action field (Karimpur et al. 2020; Fiehler and Karimpur 2023). Visuomotor transformations for goal-directed gaze and reach movements, notably when self-motion is involved, are better explained by invoking an underlying 3-dimensional rotational and translational geometry, with parameters updated in an ongoing manner, relying on memory, multimodal information, relating sensors to effectors through different frames and modulating both direction and depth relative to target in a manner that is calibrated based on motion parallax cues (Crawford et al. 2011). Such a geometry is included in 3-dimensional projective geometry. Visuomotor transformations for reaching seem to rely on an internal model of the complete eye-to-shoulder linkage geometry (Blohm and Crawford 2007).

Such a need to switch between multiple reference frames and perspectives or more generally to relate them, and to operate across space at different scales adaptively and flexibly in order to seamlessly relate perception, imaginary perspectives and action in space, is one of the motivations for the PCM.

Beyond sensory modalities, there is some evidence that the consciously reported intensity of emotional experience as a function of the distance of a stimulus in 3-dimensional space is consistent with a projective structure and perspectival appraisal of affective information, as, at least for threatening stimuli, it obeys an inverse distance law (Teghtsoonian and Frost 1982); see (Rudrauf et al. 2022a).

There is a closed-loop relationship between the world as sensed and the world as acted upon (Koenderink 2013). Beyond standard active inference or classical theories of cybernetic control based on efference copies, which all entail some sort of internal simulation, the PCM considers such closed-loops to also be embedded into the very structure of consciousness, which intrinsically makes spatial consciousness a functional tool for action and enhances fitness through perspective taking and its impact on perceived or expected epistemic and affective values. This pervasive relationship between perception and action is embodied in the PCM through the concept of 3-dimensional projective G-space, and the equivalence it establishes between action in the environment and transformations of the internal space.

Of note, in the present contribution, we assumed a fixed calibration of the projective frame and fixed metrics in order to simplify our toy model and to be able to derive analytical results for the question at hand; but a more dynamical version of the model, as in our work on the Moon Illusion (Rudrauf et al. 2020a), is certainly something that we should study in the future, including modeling mechanisms of adaptive spatial attention. We did, however, manipulate one of the projective parameters in our simulations in order to show how it directly impacts epistemic value and exploratory behaviours.

## Methodology

We first implemented a simple search task in a toy model with one object to be able to mathematically demonstrate fundamental properties of geometries on epistemic values and curiosity-driven approach behaviours. We then extended this simple task to two objects, based on a slightly modified version of the toy model, to further explore through numerical simulations the ability of the geometries considered to generate exploration behaviours.

The experiment we considered is that of an agent, denoted as *a*, which is looking for an object *O* in the ‘real world’, the 3-D Euclidean space $E_3:={\mathbb {R}}^3$. The set of moves of the agent is denoted *M*. The position of *O* is denoted $o\in E_3$. The agent ‘represents’ the position of the object *O* inside its ‘internal world model’. We consider ‘internal world models’, spaces denoted *W*, that are such that there is a group acting on them; we call such spaces, group-structured world models. This group accounts for the change of perspective that each movement of the agent induces; a perspective on the world is analogous to a choice of a (non-linear) coordinate system, when the agent moves this coordinate system changes; and the changes of coordinates are the group actions. We consider two spaces in particular:

1. *Euclidean case*: *W* is the 3-D vector space, $W= {\mathbb {R}}^3$
2. *Projective case*: *W* is the 3-D projective space, denoted as $P_3({\mathbb {R}})$

**Fig. 1:**
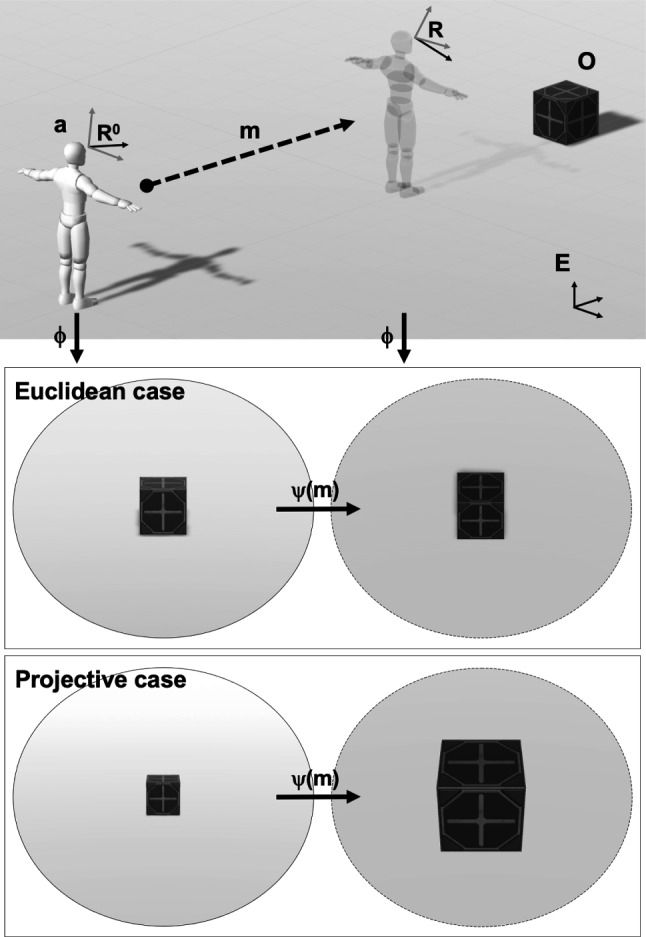
One-object toy model setup and main transformations. *Upper-tier.* Agent **a** simulates move *m* in Euclidean space *E*. ${\mathcal {R}}^0$ and ${\mathcal {R}}$ are its frames in *E* before and after the move, oriented toward object *O*. Vertical arrows indicate transformations $\phi$ from the external to the internal space. *Lower-tier.* Rendering (made with Unity) of the effect of the internal group action $\psi (m)$ corresponding to move *m* in the Euclidean versus projective case. The images for the projective case correspond to a 2-dimensional rendering of the projective space onto an image plane. In 3-dimensions, a cube would appear as a truncated pyramid, with a foreground face larger than the background face, and a compression of the distance between these faces increasing with distance from the observer; toward infinity, the cube would look like a 2-dimensional square (see examples in Rudrauf et al. 2022b). This is consistent with the perception of objects in space, e.g., in vision the Moon would appear as a large sphere to an observer close to it but as a small disk to an observer on Earth (it is practically at infinity for visual space; Rudrauf et al. 2020b)

In Sect. 3.2, we explain how the ‘real world’ and the ‘internal world model’ are related to one another in both the Euclidean and projective cases. Figure 1 illustrates the setup of the one-object toy model and main transformations considered. The agent’s internal beliefs about the position of the object are encoded by a probability measure on *W* that the agent updates through observations. The agent explores its environment through the computation of an epistemic value (mutual information), the maximization of which captures curiosity-based exploration. In Sect. 3.3, we explain how epistemic value is defined for group structured internal representations. In Sect. 3.4 we give the details of the exploration algorithm.

### Group Structured World Model

In general, groups can be seen as encoding transformations or changes of frame.

#### Definition 1

(Group, §2 Chapter 1 Lang 2012) A group is a set *G* with an operation :$G\times G\rightarrow G$ that is associative, such that there is an element $e\in G$ for which $e.g=g$ for any $g\in G$, and any $g\in G$ has an inverse denoted $g^{-1}$ defined as satisfying, $g.g^{-1}= g^{-1}.g=e$.

We call a group-structured world model, a world model provided with a group action; we now make this statement formal.

#### Definition 2

*W* is a group-structured world model for the group *G* when there is a map $h: G\times W\rightarrow W$ denoted as $h(g,x)=g.x$ for $g\in G$ and $x\in G$, such that,

1. $$(g.g_1).x= g.(g_1.x)$$ for all $$g,g_1\in G$$, $$x\in W$$
2. $$e.x=x$$, for all $$x\in W$$

The group-structured world model is usually called a *G*-space, but we will keep the former denomination for now to recall the context we consider, viz., that of an agent exploring its environment with noisy sensors. In the *Euclidean case* the group structured world model, *W*, is the 3-D vector space ${\mathbb {R}}^3$: the Euclidean space with additional information of a center and three axes, one on front, one on the right and one above; it is structured by the group of invertible affine transformations $GL_3({\mathbb {R}})\ltimes {\mathbb {R}}^3$, the semi-direct product of $GL_3({\mathbb {R}})$ acting on ${\mathbb {R}}^3$. In a formal result (Theorem 1) we only consider its subgroup *E*(3), the Euclidean group of invertible affine transformations from the space into itself preserving the Euclidean metric: it is the group that contains all possible moves of the agent, and the result holds for this group. In the simulation, we consider the smaller subgroup of 2-D affine transformations $E(2)\hookrightarrow E(3)$ as it corresponds to affine transformations on the plane surface the agent is evolving on. The 3-dimensional projective space $P_3({\mathbb {R}})$ is the set of lines in ${\mathbb {R}}^4$. As a matrix changes one line into another, it induces an action on the projective space.

In the *projective case*, the group-structured world model, *W*, is the projective space $P_3({\mathbb {R}})$; it is structured by the group of projective linear transformations $PGL_4({\mathbb {R}})$, i.e., matrices acting on ${\mathbb {R}}^4$.

### Relating the ‘Real World’ to the ‘Internal World Model’

We assume that the ‘real world’ is or is ‘in’ 3-D Euclidean space, $E_3$; it is the space that is used to set up the experiment: the conformation of the agent and of the place of the object. We assume that the ‘real world’ comes with a Euclidean frame ${\mathcal {R}}_E$, i.e., a point ${\mathcal {C}}$ and three independent vectors $e_0,e_1,e_2$. This frame is used to set up the experiment: the configurations of the object and agent across time are encoded in this frame; it is fixed once and for all before starting the experiment. Therefore we now identify $E_3$ with ${\mathbb {R}}^3$, ${\mathcal {C}}$ with (0, 0, 0) and $e_0,e_1,e_2$ with the respective basis vectors, (1, 0, 0), (0, 1, 0), (0, 0, 1). The agent, denoted as **a**, is modeled as a solid in the ‘real world’; it has its own Euclidean frame (the solid reference frame), ${\mathcal {R}}:=({\mathcal {P}},u_{0},u_{1},u_{2})$, with ${\mathcal {P}}$ the center of *a* and $u_{0},u_{1},u_{2}$ three unitary vectors that form a basis. In the *Euclidean case*, the map that relates $E_3$ and its group-structured world model, *W*, is the affine map, $\phi _{{\mathcal {R}}}$, that changes the coordinate in ${\mathcal {R}}_E$ to coordinates in ${\mathcal {R}}$. In the *projective case*, this map is a projective transformation. The choice of such a projective transformation is dictated by Proposition A.1 (Rudrauf et al. 2022b). Let us now recall some facts about the transformation dictated by Proposition A.1 (Rudrauf et al. 2022b); let for any $(x,y,z)\in {\mathbb {R}}^3$,

1 $$\begin{aligned} \rho (x,y,z)= \left( \frac{x}{\gamma z+1}, \frac{y}{\gamma z+1}, \frac{z}{\gamma z+1}\right) \end{aligned}$$

with $\gamma \in {\mathbb {R}}_{+}$ a strictly positive parameter. The (projective) transformation $\phi ^p_{{\mathcal {R}}}$, from $E_3$ to *W*, which relates the ‘real world’ to the ‘internal world model’ in the projective case, is posited to be $\phi ^p_{{\mathcal {R}}}:=\rho \circ \phi _{{\mathcal {R}}}$. It is an essential component of the PCM as this transformation is the one that will allow relating the actions of the agent in the real world to consequences in its internal projective world model.

#### Proposition 1

When the agent **a** makes the move $m\in M$, its solid reference frame changes from ${\mathcal {R}}$ to ${\mathcal {R}}^m$. In the *Euclidean case* this move induces invertible affine transformations $\psi _m\in E(3)$ from the ‘internal world model’ to itself. In the *projective case* it induces a projective transformation, $\psi _m\in PGL_4({\mathbb {R}})$.

#### Proof

Euclidean case:

The moves of the agent are translations and rotations on the surface it is evolving on; therefore, its changes of frame are dictated by 2-D affine transformations, which form a subgroup of the 3-D Euclidean group, i.e., $E(2)\hookrightarrow E(3)$.

Any 3-D affine transformation is encoded by a matrix $M=(m_{i,j}; i,j=1..3)$ and a vector $(m_{j,4}; j=1..3)$; the change of coordinates $\phi _{{\mathcal {R}}}$ is defined by the matrix $(m_{i,j}^{{\mathcal {R}}}; i,j=1..3)$ and the vector $(m_{4,j}^{{\mathcal {R}}}; j=1..3)$.

Projective case: $\phi ^p_{{\mathcal {R}}}=\rho \circ \phi _{{\mathcal {R}}}$ is the projective map with expression in homogeneous coordinates given by the matrix,

$$\begin{aligned} \begin{pmatrix} m_{1,1}& m_{1,2} & m_{1,3}& m_{1,4}\\ m_{2,1}& m_{2,2} & m_{2,3}& m_{2,4}\\ m_{3,1}& m_{3,2} & m_{3,3}& m_{3,4}\\ 0& 0 & \gamma & 1 \end{pmatrix} \end{aligned}$$

By construction, the transition map in the projective case, $\psi _m^p$, is $\phi ^p_{{\mathcal {R}}^m}\circ {\phi ^p_{{\mathcal {R}}}}^{-1}$; it is the composition of two projective transformations, therefore it is a projective transformation.

$$\square $$

We denote by ${{\,\textrm{1}\,}}_U$ or $x\rightarrow {{\,\textrm{1}\,}}[x\in U]$ the characteristic function of subset *U*, i.e., the function that is equal to 1 for $x\in U$ and 0 elsewhere.

#### Remark 1

In both cases there is a dense open subset *U* of *W* which is in continuous bijection with ${\mathbb {R}}^3$. From the Lebesgue measure *dx* on ${\mathbb {R}}^3$, we define the following measure on *W*, $d\lambda :={{\,\textrm{1}\,}}_U dx$. In what follows we do not mention this technical point further and simply refer to $d\lambda$ as the Lebesgue measure on *W*.

### Beliefs, Policies and Epistemic Value

#### Beliefs

**Beliefs:** The agent **a** keeps internal beliefs about the position of the object represented in its ‘internal world model’; these beliefs are encoded by a probability measure $Q_X\in {\mathbb {P}}(W)$, where ${\mathbb {P}}(W)$ denotes the set of probability measures on *W*. These beliefs are updated according to noisy sensory observations of the position of *O*. Sensors may introduce noise, for instance, when one focuses on a specific location in the hope of locating an object. In these situations, sensor-related uncertainty is inevitable, leading to a certain “radius" of uncertainty (typically represented by the standard deviation) around the expected object position. ‘Markov Kernels’ or stochastic maps can be used to formalize noisy sensors. Let us recall their definition. We will denote by ${\mathcal {B}}(\Omega )$ the Borel $\sigma$-algebra of the respective topological space $\Omega$.

##### Definition 3

(Markov Kernel) A ‘Markov Kernel’ $\Pi$ from $\Omega$ to $\Omega _1$ is a map $\Pi :\Omega \times {\mathcal {B}}(\Omega _1)\rightarrow [0,1]$ such that,

1. for any $\omega \in \Omega$, $\Pi (\omega ,.)$ is a probability measure over $\Omega _1$
2. $\Pi (.,A)$ is a measurable map for any $A\in {\mathcal {B}}(\Omega )$.

In particular, it is a (measurable) map that sends any $\omega _1\in \Omega _1$ to a probability measure $\Pi _{\vert \omega _1}\in {\mathbb {P}}(\Omega )$. Equivalently, when $\Omega$ and $\Omega _1$ are finite, for any $\omega _1\in \Omega _1$, $\sum _{\omega \in \Omega } P(\omega \vert \omega _1)=1$

The uncertainty stemming from the sensors of **a** is captured by a Markov Kernel $P_{Y\vert X}$ from *W* to *W*. It is a parameter of the experiment: it is fixed before the agent starts looking for *O*. The couple $(P_{Y\vert X},Q_X)$ defines the following probability density, $P_{X,Y}\in {\mathbb {P}}(W\times W)$: for any $x,y\in W$, $P_{X,Y}(dx,dy):= p_{Y\vert X}(y\vert x) q_X(x)dxdy$, where *dx* is the Lebesgue measure on *W*. An observation of the position of the object $y^{o}\in W$ triggers an update of the belief $Q_X$ to the belief with density:

2 $$\begin{aligned} Q_{X\vert y^{o}}= \frac{p_{Y\vert X}( y^{o}\vert x) q_X(x) dx}{\int _{x\in W} p_{Y\vert X}(y^{o}\vert x) q_X(x) dx} \end{aligned}$$

The former is Bayes’ rule.

#### Policies

The agent has a set of moves it can make *M*; a move $m\in M$ is associated to the action of the group element $\psi _m: W\rightarrow W$ (Proposition 1). The agent anticipates the consequences of its moves on its internal world model one step ahead: each change of frame induces the following Markov Kernel, for any $m\in M$, $A\subseteq W$ a (Borel) subset of *W*, and $x_0\in W$, $p_{X_1\vert X_0,m}(A\vert x_0, m)= {{\,\textrm{1}\,}}[\psi _m(x_0)\in A]$. Each move *m* spreads a prior $Q_X$ on $X_0$ into the following prior on $X_1$: $\forall A\in {\mathcal {B}}(W)$,

3 $$\begin{aligned} \psi _{m,*}Q_X(A)&:= \int {{\,\textrm{1}\,}}[\psi _m(x_0)\in A] q_{X}(x_0) dx = Q_X(\psi _m^{-1}A) \end{aligned}$$

**Fig. 2:**
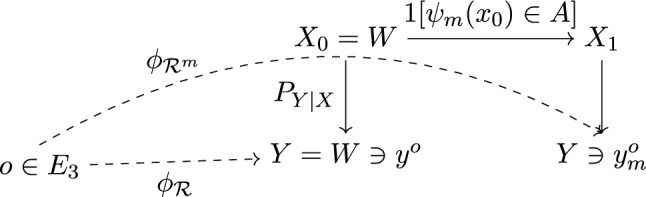
$m\in M$ is a move of the agent *a*; $1[\psi _m(x_0)\in A]$ defines the kernel induced by move *m*; $P_{Y\vert X}$ is the noisy sensor. The diagram constituted of solid arrows defines the generative model the agent uses to plan its actions. *o* is the position of the object in the ‘real world’; $y^{o}\in W$ is the representation of *o* in the ‘internal world model’ of **a** with respect to the solid reference frame ${\mathcal {R}}$; $y^{o}_m$ is the same for the reference frame ${\mathcal {R}}^m$ after move *m*

We chose to denote this probability measure as $\psi _{m,*}Q_X$, because it is the standard mathematical notation for the ‘pushforward measure’ by $\psi _{m}$. The generative model the agent uses to plan its future actions is summarized in Fig. 2.

#### Epistemic Value

The following definition is a restatement of the *epistemic value* introduced in Friston et al. (2015) applied to kernels $P_{Y\vert X}:W\rightarrow W$.

##### Definition 4

(Epistemic Value) For any probability measure $Q_X\in {\mathbb {P}}(W)$, the epistemic value of this measure is:

4 $$\begin{aligned} C(Q_X):=&{\mathbb {E}}_{P_{Y}}\left[ H(P_{X\vert Y} \vert Q_X)\right] \end{aligned}$$

5 $$\begin{aligned}&=\int p_{Y}(y)dy \int p_{X\vert Y}(x\vert y) \ln \frac{p_{X\vert Y}(x\vert y)}{q_X(x)} dx \end{aligned}$$

*H* is the relative entropy, also called Kullback–Leibler divergence.

Re-expressing Eq. 4, it becomes apparent that epistemic value is simply a mutual information:

6 $$\begin{aligned} C(Q_X)=\int p_{X,Y} \ln \frac{p_{X,Y}(x,y)}{p_Y(y)q_X(x)} dx dy \end{aligned}$$

We propose to define the epistemic value of move *m* as the epistemic value of the induced prior on $X_1$, $C(m):= C(\psi _{m,*}Q_X)$.

### Algorithm

Let us now put the previous elements together to describe the exploration behavior programmed in our agent. The agent **a** is initialized in a configuration of the ‘real world’, with solid reference frame ${\mathcal {R}}^0$; the object *O* is positioned at $o\in E_3$. **a** starts with an initial belief $Q_{X}^0\in {\mathbb {P}}(W)$ on the position of *O*. It plans one step ahead the consequence of move *m*; move *m* induces a group action $\psi _m:W\rightarrow W$ that pushes forward the belief $Q_{X}^0$ to $\psi _{m,*}Q_{X}^0$. The agent then evaluates the epistemic value of $(P_{Y\vert X},\psi _{m,*}Q_{X}^0)$ for each move *m* and chooses the move that maximizes this value, ${\overline{m}}$. **a** executes the move ${\overline{m}}$ which transforms its solid reference frame ${\mathcal {R}}_0$ to ${\mathcal {R}}$. It can then observe (with its ‘noisy sensors’) the position of *O* which is $y^{o}:=\phi _{{\mathcal {R}}}(o)$ in its internal world model, which triggers the update of prior $\psi _{{\overline{m}},*}Q_{X}^0$ to the distribution conditioned on the observation: $\left( \psi _{{\overline{m}},*}Q_{X}^{0}\right) _{\vert y^{o}_{{\overline{m}}}}$. The process is iterated with this new prior. The exploration algorithm is summarized in Algorithm 1.

**Algorithm 1:**
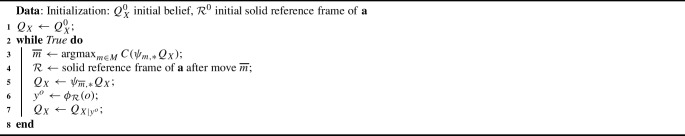
Curiosity based Exploration for agent **a**

## Theoretical Predictions

We prove that the group by which the internal world model is structured influences the exploration behavior of the agent. The *Euclidean case* serves as the reference model; in this case the world model shares the same structure as the real world: it is the ‘classical’ way of modeling this exploration problem. The *projective case* corresponds to the hypothesis underlying the PCM. We consider the following noisy sensor, for any $x,y\in {\mathbb {R}}^3$, $P_{Y\vert X}(y\vert x)= \frac{3}{4\pi \epsilon ^3} {{\,\textrm{1}\,}}[\Vert x-y\Vert \le \epsilon ]$ where $\Vert . \Vert$ designates the Euclidean norm on ${\mathbb {R}}^3$, i.e. $\Vert x\Vert ^2 = x_0^2+x_1^2+x_2^2$; $\epsilon >0$ is a strictly positive real number.

### Theorem 1

Let us assume that staying still is always a possible move for the agent.

*Euclidean case:* when the agent has an objective representation of its environment, given by an affine map, the agent stays still.

*Projective case:* Assume now that the set of moves *M* is finite; assume furthermore that after any possible move, the agent faces *O*, in other words, we assume that the agent knows in which direction to look in order to find the object but is still uncertain on *where* the object is exactly. If it has a ‘subjective’ perspectives, i.e., its representation is given through a projective transformation, it will choose the moves that allows it to approach *O* (for any $\epsilon$ small enough).

### Proof

Let us first give an idea of the proof. The agent circumscribes a region of space in which it believes it is likely to find the object. This region corresponds to the error the agent tolerates on the measurement it makes of the position of *O*; we can also see it as the precision up to which the agent measures the position of *O*. In the *Euclidean case*, the region in which the agent circumscribes the object appears to always be of the same size, irrespective of the agent’s configuration with respect to the object. Therefore not moving ends up being an optimal option and the agent will not approach the object without additional extrinsic reward. In the *projective case*, the agent can ‘zoom in’ on this region in order to gain more precision in measuring *o*; the configurations of the agent in which this region is magnified are more informative regarding the position of *O* and therefore preferred by the agent. The only way for the agent to actually zoom in on this area is to approach the location it believes *O* is likely to be; therefore the agent will end up approaching *O*.

Let us now give the details of the proof. We will denote $B_y^{\epsilon }$ the Euclidean ball of radius $\epsilon$ around $y\in {\mathbb {R}}^3$, i.e. $B_y^{\epsilon }=\{ x\in {\mathbb {R}}^3 \vert \quad \Vert x-y\Vert \le \epsilon \}$.

### Lemma 1

For any $Q\in {\mathbb {P}}(W)$, both in Euclidean and projective cases, for any affine map or projective transformation $\psi :W\rightarrow W$,

7 $$\begin{aligned} C(\psi _{*}Q)=-\int dy Q(\psi ^{-1}( B_y^{\epsilon })) \ln Q(\psi ^{-1}( B_y^{\epsilon })) \end{aligned}$$

### Proof

8 $$\begin{aligned} C(\psi _{*}&Q)= \frac{3}{4\pi \epsilon ^3}\times \nonumber \\&\int \psi _{*} Q(d x_1) \int dy {{\,\textrm{1}\,}}[x_1\in B_y^{\epsilon }] \ln \frac{{{\,\textrm{1}\,}}[x_1\in B_y^{\epsilon }] }{\int \psi _{*} Q(d x_1) {{\,\textrm{1}\,}}[x_1\in B_y^{\epsilon }]} \nonumber \\&=-\frac{3}{4\pi \epsilon ^3}\int dy \ln Q(\psi ^{-1}(B_y^{\epsilon })) \int \psi _{*} Q(d x_1){{\,\textrm{1}\,}}[x_1\in B_y^{\epsilon }] \nonumber \\&= -\frac{3}{4\pi \epsilon ^3}\int dy Q(\psi ^{-1}( B_y^{\epsilon })) \ln Q(\psi ^{-1}( B_y^{\epsilon })) \end{aligned}$$

$$\square $$

*Back to the proof of the Theorem:*

Euclidean case: for any set of moves *M*, and for any $m\in M$, $\psi _m$ is a displacement; therefore for any $y\in W$, $\psi ^{-1}_m(B_y^{\epsilon })=B_{\psi ^{-1}_m(y)}^{\epsilon }$. Then, for any prior $Q\in {\mathbb {P}}(W)$,

9 $$\begin{aligned} C(\psi _{m,*}Q)&=- \frac{3}{4\pi \epsilon ^3}\int dy Q(B_{\psi ^{-1}_{m}(y)}^{\epsilon })\ln Q(B_{\psi ^{-1}_{m}(y)}^{\epsilon }) \nonumber \\&=-\frac{3}{4\pi \epsilon ^3}\int dy Q(B_{y}^{\epsilon })\ln Q(B_{y}^{\epsilon }) \end{aligned}$$

In this case, the epistemic value is independent from the change of Euclidean frame, and not moving is a perfectly valid choice for the agent to maximize it, at each time step of the exploration algorithm (Algorithm 1).

The fact that staying still is a valid strategy arises as the agent assumes (or believes) that it has access to the whole configuration space of *O*. If it knew it had limited access to it, through, for example, limited sight, we expect the agent would look around until the object *O* would be in sight and then stop moving (see below two-object simulations for a numerical demonstration of such behaviour).

Projective case: Consider two projective transformations $\psi ,\psi _1:W\rightarrow W$, if for any $y\in W$,

10 $$\begin{aligned} \psi ^{-1}(B_y^{\epsilon })\subseteq \psi _1^{-1}(B_y^{\epsilon }) \end{aligned}$$

then,

11 $$\begin{aligned} -Q(\psi ^{-1}(B_y^{\epsilon }))&\ln Q(\psi ^{-1}(B_y^{\epsilon })) \end{aligned}$$

12 $$\begin{aligned}&\ge -Q(\psi _1^{-1}(B_y^{\epsilon }))\ln Q(\psi _1^{-1}(B_y^{\epsilon })) \end{aligned}$$

for $Q(\psi ^{-1}_1(B^{\epsilon }_y))$ around 1 (bigger than 1/*e*).

This suggests that the moves that maximize epistemic value are those where $\psi ^{-1}_m$ shrinks the zone around $y^{o}=\rho (\phi _{{\mathcal {R}}}(o))$, which is the representation of *O* in the internal world of the agent. In particular, it means magnifying the zone around $\rho (\phi _{{\mathcal {R}}^m}(o))$ in the agent’s new frame, ${\mathcal {R}}^m$, after move *m*. The only way to do so is to select moves that bring the agent closer to *O*. Let us denote $y^{o}_m:= \rho (\phi _{{\mathcal {R}}^m}(o))$. Let us now make the previous argument more formal. We assume that the set of moves *M* is finite. Let $Q_0=q_0 d\lambda$ be any initial prior on $W=P_3({\mathbb {R}})$, at stating time $t=0$. After one step, move $m_1$ is chosen and the agent updates its prior as,

13 $$\begin{aligned} q_1(x)\propto 1[x\in B_{y^{o}_{m_1}}^{\epsilon }]q_0(x) \end{aligned}$$

where $\propto$ means ‘proportional to’. The prior we now consider is $Q_1$ denoted simply as *Q*. One shows that there is $\alpha >0$, such that for all $m\in M$, and $\epsilon >0$ small enough,

14 $$\begin{aligned} C(&\psi _{m,*}Q)= -\frac{3}{4\pi \epsilon ^3}\times \end{aligned}$$

15 $$\begin{aligned}&\int dy {{\,\textrm{1}\,}}[y \in B_{y^{o}_m}^{\alpha \epsilon }] Q(\psi ^{-1}_m( B_y^{\epsilon })) \ln Q(\psi ^{-1}_m( B_y^{\epsilon })) \end{aligned}$$

Let $\approx$ stand for ‘approximately equal to’ (equal at first order in development in powers of $\epsilon$). Then from the previous statement the summand can be approximated by its value in $y^{o}_m$:

16 $$\begin{aligned} C(\psi _{m,*}Q)\approx -\alpha ^3 Q(\psi ^{-1}_m( B_{y^{o}_m}^{\epsilon })) \ln Q(\psi ^{-1}_m( B_{y^{o}_m}^{\epsilon })) \end{aligned}$$

Furthermore, $Q(\psi ^{-1}_m( B_{y^{o}_m}^{\epsilon })) \approx \frac{4\pi \epsilon ^3}{3} \frac{q_1(y^{o})}{\vert \det \nabla \psi _m\vert (y^{o})}$, where $\vert \det \nabla \psi _m\vert (y^{o})$ is the absolute value of the Jacobian determinant of $\psi _m$ at $y^{o}$. The epistemic value is maximized when $\vert \det \nabla \psi _m\vert (y^{o})$ is maximized. By definition, $\psi _m= \rho \circ \phi _{{\mathcal {R}}^m}\circ \phi _{{\mathcal {R}}}^{-1}\circ \rho ^{-1}$, therefore, by the chain rule of differentiation

17 $$\begin{aligned}&\vert \det \nabla \psi _m\vert (y^{o}) \nonumber \\&= \vert \det \nabla \rho \vert (\phi _{{\mathcal {R}}^m}(o)). \vert \det \phi _{{\mathcal {R}}^m}\vert (o). \vert \det \nabla [\phi _{{\mathcal {R}}}^{-1}\circ \rho ^{-1}]\vert (y^{o}) \end{aligned}$$

Let us make explicit each terms in the previous equation. $\phi _{{\mathcal {R}}^m}$ is a rigid movement therefore, $\vert \det \phi _{{\mathcal {R}}^m}\vert (o)=1$. $\vert \det \nabla [\phi _{{\mathcal {R}}}^{-1}\circ \rho ^{-1}]\vert (y^{o})$ does not depend on *m*, so we can label it as a constant *C*. $\phi _{{\mathcal {R}}^m}(o)$ is the coordinate of *O* in the Euclidean frame ${\mathcal {R}}^m$; let us denote by $(x^m,y^m,z^m)$ these coordinates, i.e., $(x^m,y^m,z^m):=\phi _{{\mathcal {R}}^m}(o)$. Then,

18 $$\begin{aligned} \vert \det \nabla \rho \vert (x^m,y^m,z^m)= \frac{1}{(\gamma z^{m}+1)^4} \end{aligned}$$

Therefore, $$\vert \det \nabla \psi _m\vert (y^{o})= C^{'}\frac{1}{(\gamma z^{m}+1)^4}$$.

As we assumed that for any move $m\in M$, the object *O* is always in front of the agent, then $z^{m}\ge 0$; in this case, $z^{m}$ is also the distance of the agent to the object. Epistemic value is maximized when $z^{m}$ is minimized and therefore the agent selects moves that reduce its distance to the object. Denote one of such move ${\overline{m}}$; the argument then loops back with the new reference frame ${\mathcal {R}}^{{\overline{m}}}$ and updated belief $Q\leftarrow \psi _{{\overline{m}},*} Q_{\vert y^{o}_m}$. $\square$

In the next section we present an implementation of this experiment and simulation results for both the one-object setup and a two-object setup with limited field of view.

## Simulations

### One-object Simulation Methods

Algorithm 1 is implemented in the following manner. Beliefs and the Markov Kernel corresponding to sensors were considered to be multivariate normal distributions, that is $P_{Y\vert X} \sim {\mathcal {N}}(\mu _{Y \vert X},\,\Sigma _{Y \vert X})$ and $Q_X \sim {\mathcal {N}}(\mu _X,\,\Sigma _X)$. Belief update through the action of a group was approximated using a Gaussian distribution; a projective transformation changes a Gaussian distribution into a non-Gaussian one which is difficult to describe. Therefore we replace this non-Gaussian distribution with a Gaussian distribution with same mean and variance. We assumed $\mu _{Y \vert X} = x$ (which implies $\mu _y=\mu _x$) and $\Sigma _{Y \vert X} = \epsilon ^2{\mathbb {I}}$ where ${\mathbb {I}}$ is the identity matrix and $\epsilon >0$ a positive real number. As a result, for a given observation $y^o$, $Q_{X\vert {y^o}}$ and $C(\psi _{m,*} Q_X)$ can be computed efficiently. The joint distribution *P* on *X*, *Y* is a Gaussian distribution:

$$\begin{aligned}P(x,y) = p(y\vert {x})p(x)\\P(x,y) \sim {\mathcal {N}}(\mu _{X,Y},\,\Sigma _{X,Y})\end{aligned}$$

with $$\mu _{X,Y} = (\mu _X, \mu _X)$$ and

19 $$\begin{aligned} \Sigma _{X,Y} = \begin{pmatrix} \Sigma _{XX} & \Sigma _{XX}\\ \Sigma _{XX}& \epsilon ^2{\mathbb {I}}+\Sigma _{XX} \end{pmatrix} \end{aligned}$$

The variance of *Y* is $\Sigma _{YY} = \epsilon ^2{\mathbb {I}}+\Sigma _{XX}$.

The joint distribution being Gaussian entails that the distribution of *X* conditioned on $y=y^o$ is also Gaussian, thus $Q_{X\vert {y^o}} \sim {\mathcal {N}}(\mu _{X\vert {y^o}},\,\Sigma _{X\vert {y^o}})$. Applying Proposition 3.13 (Eaton 2007) to our setting, the mean and covariance of the conditioned distribution are given by:

20 $$\begin{aligned} &  \mu _{X\vert {y^o}} = \mu _X + \Sigma _{XX} \Sigma _{YY}^{-1} (y^o - \mu _X) \end{aligned}$$

21 $$\begin{aligned} &  \Sigma _{X\vert {y^o}} = \Sigma _{XX} - \Sigma _{XX}\Sigma _{YY}^{-1}\Sigma _{XX} \end{aligned}$$

Epistemic value is computed using the Kullback–Leibler divergence. With full knowledge of the joint distribution, in the Gaussian case, following the expression of entropy for Gaussian vectors (Chapter 12 Equation 12.39, Cover and Thomas 2006) it is computed as:

22 $$\begin{aligned} C(Q_X) = I(X; Y) = \frac{1}{2} \ln {\frac{ (\det {\Sigma _{XX}})(\det {\Sigma _{YY}}) }{\det {\Sigma _{X,Y}}}} \end{aligned}$$

The set of moves that can be selected by the agent is restricted to translations as the agent must always face the object. The set of possible translations is composed of eight translations with the same norm, with evenly distributed angles (one of them being oriented toward the object irrespective of the position of the agent), and also contained an idle state, i.e., no translation. Here the angles correspond to the angles of the translation and not a rotation angle of the solid frame of the agent, as the agent always faces the object.

We approximated the belief after the action *m* of a given group using a Gaussian distribution, $\psi _{m,*} Q_X \sim {\mathcal {N}}(\mu _{m},\,\Sigma _{m})$. The mean and covariance matrix are approximated using numerical integration:

23 $$\begin{aligned} \mu _m= &  \int {xp(\psi _m^{-1}(x)){\frac{1}{\vert \det J_{\psi _m}(\psi _m^{-1}(x)) \vert }}dx} \end{aligned}$$

24 $$\begin{aligned} \Sigma _m= &  \int {(x - \mu _m)(x - \mu _m)^{T}p(\psi _m^{-1}(x)){\frac{1}{\vert \det J_{\psi _m}(\psi _m^{-1}(x)) \vert }}dx} \end{aligned}$$

**Fig. 3:**
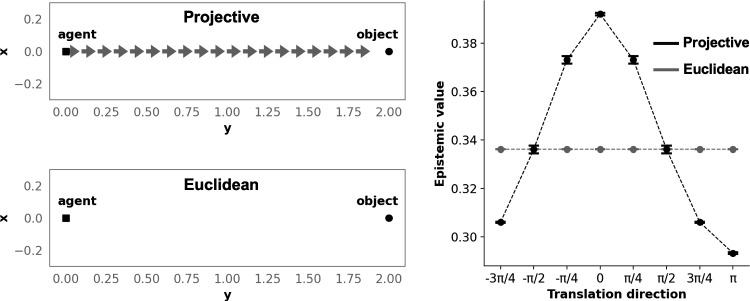
Simulation results. *Left-tier.* Trajectory of the agent for the projective versus Euclidean internal spaces. *Right-tier.* Epistemic value as a function of directions of translation with respect to the object direction, for the projective versus Euclidean internal spaces. Points are average values across comparable directions, and error-bars are standard errors

We ran two sets of simulations. In the first one (Fig. 3 left tier), the agent started from an initial position with the object always located at a fixed position, and the algorithm was applied for 20 iterations, for both the Euclidean and projective internal spaces. The agent started at (0, 0) and the object was located at (0, 2) in the world frame $E_2$ spanning the agent’s displacement floor. If all translations were associated with epistemic values that only varied within a small range ($\pm 1e-4$) as compared to the epistemic value of the idle state, reflecting numerical imprecision, the idle state was selected (the agent did not move). The aim of this set of simulations was to compare trajectories of agent displacements through time across the two geometries. In the second set of simulations (Fig. 3 right tier), the agent started at the center of a $20\times 20$ grid of possible positions of the object. The positions that are too close to the agent are excluded so that a sufficient effect can be observed. For each object position, we considered only the first set of translations that the agent could envision from its initial position. We computed epistemic value across the set of possible translations of the agent for each object position and for both the Euclidean and projective internal spaces. The aim of this set of simulations was to be able to systematically compare epistemic values across the two geometries for all possible positions of objects.

### Results

Figure 3 (left tier) shows a representative example of trajectories obtained in the Euclidean versus projective cases. In the projective case, the agent always approached the object. In the Euclidean case, the agent always stayed idle. Figure 3 (right tier) shows epistemic value as a function of translation direction expressed in radians for both the projective and Euclidean cases. The direction of 0 radians corresponds to the object direction. We averaged epistemic values across object positions within comparable directions. In the projective case, average epistemic value was maximal for the direction of the object and decreased with directions farther away from it. In the Euclidean case, average epistemic value was identical across directions. Maximum average epistemic value was much higher for the projective case than for the Euclidean case.

### Two-object setup

An extension of Algorithm 1 was implemented for two-object simulations to further study how the models may induce exploration behaviours in a more complex landscape and setup with limited fields of view. The agent *a* keeps independent internal beliefs $Q_{X_i}$ about the position of each object $O_i \in {\mathbb {O}}$, where ${\mathbb {O}}$ is the set of objects. An object $O_i$ is positioned at $o_i \in E_2$. An observation can be obtained for each object, and the corresponding beliefs are updated as described in Algorithm 1.

We extended the actions of the agent to include rotations, changing how the agent may be oriented with respect to the two objects. We defined a field of view for the agent, which acted as a mask on the world. Every object included in the field of view yielded an observation that was used to update the agent’s beliefs about the position of the object. Beliefs about an object that was not in the field of view were not updated, $Q_{{X_i} \vert y^{o_i}} = Q_{X_i}$. Note that, to simplify the algorithm and reduce simulation time, the internal representation space with respect to each object was still oriented towards the object considered (as if the agent imagined it was facing a given object), and that rotations only impacted the field of view.

At each time step, the agent selected its optimal orientation and translation. The epistemic value of an action was the sum of the epistemic values $C(Q_{X_i})$ of these actions relative to each $Q_{X_i}$. When the object was not in the field of view, there were no possible observations, so the uncertainty of the observation is total: it is a uniform law on the possible configurations of that object. Following Pearl’s update rule (Jacobs 2019), the associated updated belief is $q^{up}_{X_i}(x)=\frac{\int dy\ p_{Y_i\vert X_i}(y\vert x) q_{X_i}(x)}{\int \int dy dx_1\ p_{Y_i\vert X_i}(y\vert x_1) q_{X_i}(x_1)}$, and $Q^{up}_{X_i}=q^{up}_{X_i}(x) dx$. Therefore $H(Q^{up}_{X_i} \vert Q_{X_i}) = 0$, as such $C(Q_{X_i}) = 0$.

We explored how different parameters yielded different behaviors i.e., how the agent approached different objects. The covariance of the initial prior about each object was $k{\mathbb {I}}$ with $k = 0.01$.

The first parameter was $\gamma$ as defined in 3.2, a projective parameter that may be intuitively seen as acting on how close the plane at infinity is to the origin of the projective space (acting somewhat like 3-D focal lens). As $\gamma$ approaches 0, the deformation due to projective geometry weakens. At $\gamma = 0$, the resulting space is equivalent to an Euclidean space, see Eq. (1). The second parameter we studied is $\epsilon$ as defined in 5.1. A higher $\epsilon$ entails less informative observations, as $P_{Y \vert X}$ has a higher covariance.

Let’s consider the epistemic value of the action that translates the agent toward an object in the projective case ($\gamma \ne 0$). The closer the agent is to the object, the greater the epistemic value of this action is; therefore the agent is drawn to the nearest objects. This effect is accentuated depending on the value of $\gamma$. Additionally, the epistemic value of the action decreases over time as the agent updates its beliefs after an observation. The rate at which the epistemic value decreases depends on how informative the observation is (value of $\epsilon$). The values of $\gamma$ and $\epsilon$ result in the epistemic value of an action increasing or decreasing as the agent approaches the object and receives observations about its position.

We ran simulations for $$\gamma \in [0, 1]$$ and $$\epsilon \in [0.1, 1.0]$$, to explore how these parameters interact and their effect on the behaviours of the agent in an exploration setting. The agent started at position $$a = (0,0)$$ in the world. It kept beliefs about two equidistant objects $$O_1$$, $$O_2$$, at positions $$o_1 = (-0.5,0.75)$$ and $$ o_2 = (0.5, 0.75)$$. The agent had a narrow field of view so that it could only observe the object it directly faced at a given time step.

### Exploration Behaviors

**Fig. 4:**
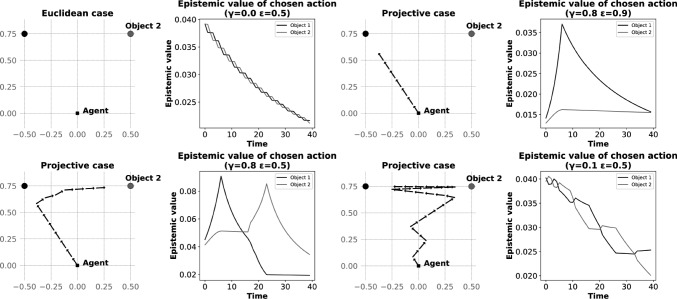
Sample trajectories in the two-object setting. Samples of trajectories (over 40 steps) of the agent for different internal spaces in the two-object setup (left tier), and epistemic value of each object for the selected action at each time step (right tier). The agent had a narrow field of view which allowed it to observe one object at a time. Observations decreased the epistemic value associated with the observed object, which resulted in the agent eventually switching direction. Different values of $\gamma$ and $\epsilon$ impacted the rate at which the agent changed direction. In the Euclidean case, which is a limit of the projective case with $\gamma = 0$, the agent did not approach either object but oriented itself alternatively toward each of them

Figure 4 shows the behaviour of the agent for sampled values of $\gamma$ and $\epsilon$. At each time step, the agent selected an action that maximized the sum of the resulting epistemic values associated with the objects. As the agent made an observation about a given object, the epistemic value of moving toward this object decreased. The priors about unobserved objects did not change; therefore the agent eventually switched its focus toward a less observed object with higher potential epistemic value. This effect was counter-balanced by the increase in epistemic value induced by geometric deformations, when the agent approached the observed object. The effects of $\gamma$ and $\epsilon$ on the agent’s oscillation between each object had a frequency that depended on the interplay between these parameters.

**Fig. 5:**
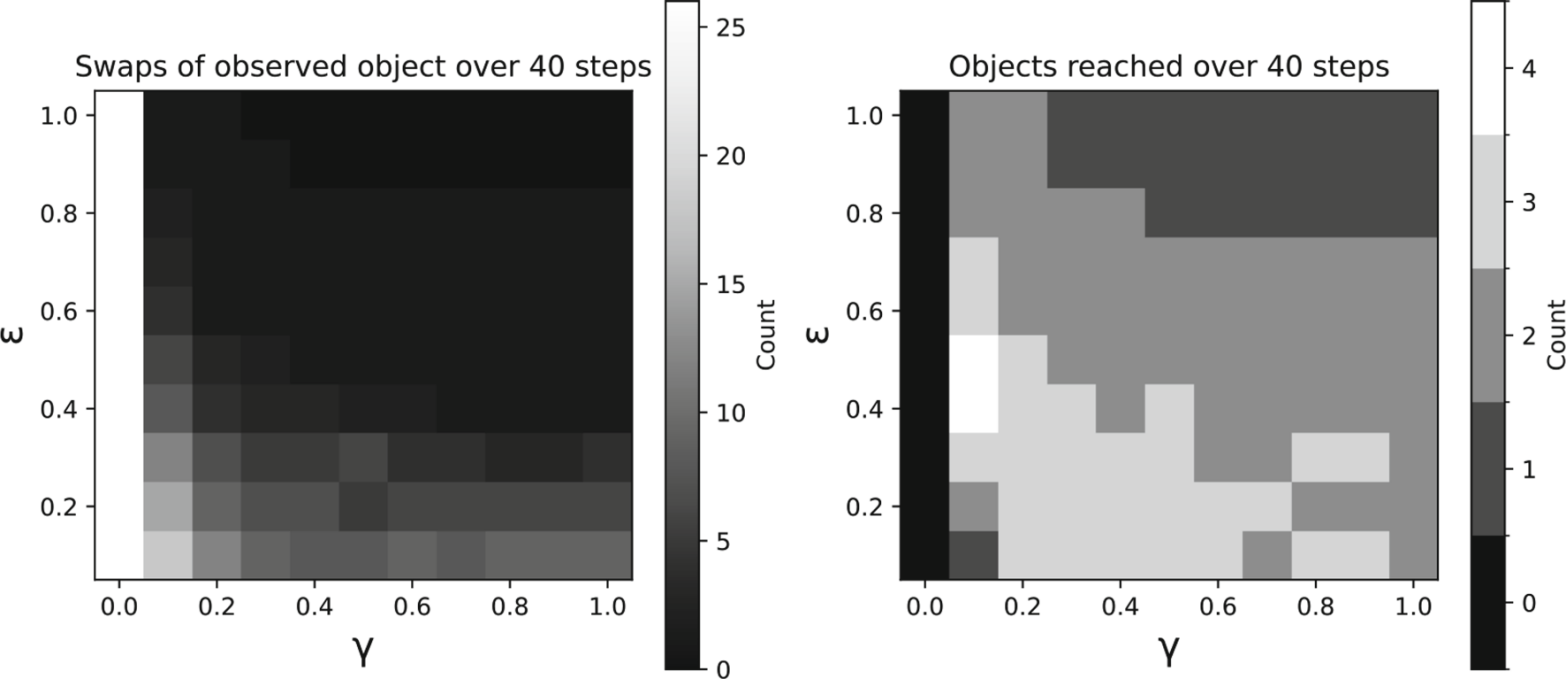
Effect of $\gamma$ and $\epsilon$ on exploration behaviour. Number of times the agent swaps orientation between objects it observes (left tier), and number of objects reached by the agent (right tier), over 40 simulation steps. An object may be reached multiple times if the agent approaches it more that once

Figure 5 characterizes the behaviour of the agent as a function of the values of $\gamma$ and $\epsilon$, in terms of both the number of swaps (changes of the agent’s orientation) between the observed objects and the number of objects reached, over constant simulation duration (the latter number can be higher than two since the agent can orient toward and approach each object more than once).

For the number of swaps, lower values of $\epsilon$ resulted in more informative observations, leading to a higher frequency of oscillations (more swaps between observed objects). Higher values of $\gamma$ resulted in a stronger drive to move toward the observed object, and therefore to a lower frequency of oscillations. Though the agent was not moving in the Euclidean case ($\gamma = 0$), it demonstrated a high frequency of swaps between objects.

We quantified aspects of the agent’s exploratory behaviour by counting the number of times the agent reached the objects (i.e., couldn’t approach them anymore without overstepping them). Parameters associated with higher swap frequency tended to lead to an increase in the number of objects reached. However, the cases of highest swap frequency resulted in a lower visit count, as the agent could not reach the observed target before switching direction. In the Euclidean case, the agent could not reach any object due to the lack of drive to move toward the objects.

The effect of projective geometry in this setting could be interpreted as modulating the extent to which the agent maintained its attention on one target, from underfocused to overfocused states leading to erratic versus sustained behaviours of approach and exploration. This resulted in exploratory behaviours that could be more or less optimal, depending on projective parameters in interaction with sensor acuity-related parameters. In the Euclidean case, the agent had no drive to approach the observed targets, and showed the highest number of swaps between objects, i.e., no sustained attention, irrespective of the acuity of the sensor.

## Discussion

In this article, we introduced a generative model for environment exploration based on a first-person perspective in which actions are encoded as changes in perspective. The families of geometry for the world model encoded possible ‘kinds’ of perspective-taking on the environment and structured the representation of sensory evidence within the world model of the agent. In other words, each family corresponded to a specific perception and imagination scheme for the agent. We encoded two such families, namely the Euclidean versus projective group as acting on the internal world model of the agent, i.e., within the geometric properties of this internal world model. We showed that different geometries induce different behaviors, focusing on the two cases: when the internal world model of the agent followed Euclidean geometry versus projective geometry. We also showed how projective and sensor-acuity parameters impacted agents’ attentional focus and patterns of exploration. These results contribute to understanding how integrative geometrical processing and principles can play a central role in cybernetics. In our approach, the geometry of the world model links perceptual and imaginary representations with actions and behaviors.

### Relation to Variational Laplace and to Free Energy

The Variational approximation of the posterior belief after observation consists of approximating the posterior with a Gaussian distribution. Variational Laplace, as discussed in Friston et al. (2007), is characterized by a Gaussian approximation of the posterior within a variational framework, for which the Laplace approximation holds precisely. To generate our simulations, we computed the mean and variance of the belief after a choice of action to approximate this belief. Doing so corresponds to performing variational inference by minimizing the Gibbs free energy over the parametric space of Gaussian distributions. We then took the posterior, with respect to the observation, of this Gaussian approximation. Therefore, our method closely relates to Variational Laplace but differs with respect to Variational Laplace as we perform the variational inference before computing the posterior.

In our setting, the second step of the active inference algorithm from the Free Energy Principle (FEP), which consists in maximizing preference through minimization of free energy, is reduced to maximizing epistemic value as defined by expected information gain. The agent only has in its memory the observation that occurred just one step before, as it keeps track of the updated belief. In our case, the agent does not keep track of previous observations, making the time series of its beliefs Markovian. In active inference, the path integral formulation of active inference (Friston et al. 2023) allows for testing the goodness of a generative world model. In our setting, the epistemic value can play this role.

### Additional Outlooks

Although beyond the scope of this article, we are interested in generalizing the approach to compare the results obtained with those that could be obtained with other models in which geometry plays a central integrative role, e.g., as in Ferreira et al. (2013) who used an internal 3-D egocentric, spherical representation of space. Such an approach would need to be expressed in terms of group action to make it formally comparable to our approach. Likewise, it would be interesting to compare other groups, and other more realistic models of sensors, and what types of behaviors they would induce. This would be useful for refining predictions and envisioning experimental designs for empirical validation in humans. We would also like to use more sophisticated settings (see for instance Rudrauf et al. 2022b, 2023); even though it may be incompatible with the derivation of analytical solutions, it could lead to richer simulations and induction of behaviors.

We also wish to further examine how the geometry of a latent space intertwines with information processing. One motivation is theoretical, as we would like to assess how geometry changes learning behavior (Goyal and Bengio 2022). In this contribution, we have discarded representation learning per se, as it was beyond the scope of this study focusing on planning. In future work, we intend to use deep learning to learn group structured representations. It is important to note that such an approach differs from geometric deep learning (Bronstein et al. 2021; Sergeant-Perthuis et al. 2022), as we do not seek to learn equivariant representations: a group structure will only be considered for the internal world model, but none will be presupposed on the observation side.

Likewise, we are interested in further examining how geometry may play a role in overt and covert attention, and more generally contextual salience. The results of our simulations demonstrated a strong link between projective parameters and attentional focus, leading to different patterns of exploration. The ability to optimally sample the environment through approach and exploration behaviours driven by curiosity is essential for agent to learn reward and punishment outcomes related to interactions with objects (and other agents) in the environment. Along these lines of inquiry, it will be important to extend the framework introduced in this contribution by integrating priors (and their updating) not only about how certain an agent is about the spatial features of objects, but also about preferences it may develop toward those objects. This should allow us to study how the geometry of the internal world model can modulate the balance between exploration and exploitation in adaptive agents following the principles of active inference with rigorous, principled mathematical formulations of models.

Our contribution could also simplify the design of novel agent architectures in which exploratory and sensory choices of actions naturally emerge as a consequence of their internal embodied representation. Likewise, another motivation for this research is more practical, as we would like to use such principles to design virtual and robotic artificial agents mimicking human cognition and behaviors following (Rudrauf et al. 2022b, 2023).

Finally, the results of this contribution add further support to the relevance of the Projective Consciousness Model.
